# Non-dialysis dependent chronic kidney disease is associated with high total and out-of-pocket healthcare expenditures

**DOI:** 10.1186/s12882-016-0432-2

**Published:** 2017-01-05

**Authors:** Christina Small, Holly J. Kramer, Karen A. Griffin, Kavitha Vellanki, David J. Leehey, Vinod K. Bansal, Talar W. Markossian

**Affiliations:** 1 Department of Public Health Sciences, Loyola University Chicago, 2160 S. First Ave, Maywood, IL 60153 USA; 2 Division of Nephrology and Hypertension, Loyola University Medical Center, Maywood, IL USA; 3 Hines Veterans Affairs Medical Center, Hines, IL USA

## Abstract

**Background:**

Previous studies have documented the high costs of non-dialysis dependent chronic kidney disease (CKD) but out-of-pocket healthcare expenditures remain poorly explored. This study described total direct and out-of-pocket expenditures for adults with non-dialysis dependent CKD and compared expenditures with those for cancer or stroke.

**Methods:**

This study used data from the 2011–2013 Medical Expenditure Panel Survey, a national survey of healthcare expenditures in the U.S. population. Expenditures were determined for adults with the following chronic diseases: CKD defined by 585 ICD9 codes (*n* = 52), cancer (colon, breast or bronchus/lung) (*n* = 870), or stroke (*n* = 1104). These represent adults who were aware of their conditions or visited a healthcare provider for the condition during the study period. Generalized linear models were used to estimate the marginal effects of CKD, cancer or stroke on adjusted expenditures compared to adults without CKD, cancer or stroke (*n* = 72,241) while controlling for demographics and co-morbidities and incorporating the sample weights of the complex survey design.

**Results:**

The mean age for group with CKD, cancer or stroke was 65.5, 66.1, and 68.2 years, respectively, while mean age for group without CKD, cancer or stroke was 47.8 years. Median values of total direct and out of pocket healthcare expenditures ranged from as high as $12,877 (Interquartile Range [IQR] $5031-$19,710) and $1439 ($688–$2732), respectively, with CKD, to as low as $1189 (IQR $196-$4388) and $226 (IQR $20-$764) in the group without CKD, cancer or stroke. After adjusting for demographics and comorbidities, the adjusted difference in total direct healthcare expenditures was $4746 (95% CI $1775-$7718) for CKD, $8608 (95% CI $6167-$11,049) for cancer and $5992 (95% CI $4208-$7775) for stroke vs. group without CKD, cancer or stroke. Adjusted difference in out-of-pocket healthcare expenditures was highest for adults with CKD ($760; 95% CI 0-$1745) and was larger than difference noted for cancer ($419; 95% CI 158–679) or stroke ($246; 95% CI 87–406) relative to group without CKD, cancer or stroke.

**Conclusions:**

Total and out of pocket health expenditures for adults with non-dialysis dependent CKD are high and may be equal to or higher than expenditures incurred by adults with cancer or stroke.

**Electronic supplementary material:**

The online version of this article (doi:10.1186/s12882-016-0432-2) contains supplementary material, which is available to authorized users.

## Background

More than 20 million people or approximately 10% of U.S. adults currently have non-dialysis dependent chronic kidney disease (CKD), [1, 2] and almost one out of every two adults aged 30–64 years is expected to develop CKD during their lifetime [3, 4]. While dialysis dependent CKD accounts for only 0.5% of the U.S. population, fee-for service expenditures for Medicare beneficiaries with dialysis dependent CKD exceeded 30 billion dollars in 2013, or over 7% of Medicare paid claims cost [5]. Nevertheless, an escalation in healthcare expenditures associated with CKD starts prior to requirement for dialysis and treatment costs escalate as non-dialysis dependent CKD progresses [6–8].

The escalation in costs in CKD is largely due to the increasing burden of comorbidities as CKD progresses [6, 9–12], thus requiring patients with CKD to seek care from multiple providers, with an average of 10.8 physician visits per year [13]. Only cancer patients had a higher average number of annual physician visits [14]. The total number of medications among adults with CKD may also be higher than most other chronic medical conditions with one study reporting that over 60% of adults with stage 3 CKD taking 5 or more different medications daily [11]. The high total number of physician visits and medications required for CKD care drives up total direct healthcare expenditures and likely also increases out-of-pocket expenditures, creating a financial burden for patients.

While total expenditures for dialysis dependent and non-dialysis dependent CKD have been previously documented, [5, 6, 15, 16] out of pocket costs for non-dialysis dependent CKD remain poorly explored. Higher out-of-pocket cost burden can impede efforts to prevent disease progression. Previous research has shown that some patients opt to not fill prescriptions or take less than the prescribed amount due to out of pocket costs [17]. The aim of this study was to describe the total healthcare expenditures including out-of-pocket costs for non-dialysis dependent CKD and compare these expenditures with those incurred for cancer and stroke in the U.S. adult population. We selected these chronic conditions because both stroke and CKD are the costliest conditions for Part A Medicare beneficiaries, while for Part B Medicare beneficiaries, cancer and CKD are the costliest conditions [18]. We hypothesized that both direct and out-of-pocket healthcare expenditures in adults with non-dialysis dependent CKD are comparable if not higher than expenditures incurred for cancer or stroke.

## Methods

### Study population

This cross-sectional study described total and out-of-pocket health care expenditures for the chronic conditions: nondialysis dependent CKD, cancer, and stroke, each condition exclusively, and compared the expenditures for these chronic conditions with those incurred among the population without CKD, cancer or stroke. The study was approved by the Loyola University Medical Center Institutional Review Board. Data were obtained from the household, medical conditions and medical provider component (MPC) files of the Medical Expenditures Panel Survey (MEPS) for years 2011 to 2013. MEPS is a household survey of the noninstitutionalized civilian population and is conducted annually. The household component provides information on respondents’ health status, demographic and socioeconomic characteristics, employment, access to care, and satisfaction with health care. For the MPC, a sample of medical providers are contacted to obtain information that household respondents can not accurately provide about dates of visits, diagnosis and procedure codes, charges and payments. The MEPS data are weighted to produce estimates of healthcare expenditures that are representative of the non-institutionalized civilian population.

The medical conditions file contains information describing current medical conditions reported by respondents during participant interviews. Information that cannot be accurately provided by participants is collected through telephone calls to providers. A current condition is defined as a condition linked to an event or disability day as well as any condition(s) the person is currently experiencing. These conditions are recorded by the interviewer as verbatim text and then translated into International Classification of Diseases, 9^th^ Edition, Clinical Modification (ICD-9-CM) codes by professional coders. To preserve confidentiality, nearly all of the diagnosis condition codes provided on this file have been collapsed to 3-digit code categories. MEPS data provide clinical classification codes (CCC) that are clinically meaningful categories that group similar conditions and are detailed in Table 1. Dialysis utilization during a given year is also collected and classified as a medical event code. Respondents reported a specific condition as being bothersome during the study period or as the reason for a medical event (hospital stay, outpatient visit, emergency room visit, home health episode, prescribed medication purchase, or medical provider visit). Therefore conditions that are undiagnosed, not bothersome during the study period, or not linked with a medical event are not ascertained in MEPS.

**Table 1:** Definition of the clinical classification software (CCS) diagnosis categories in the Medical Expenditure Panel Survey

CCS diagnosis categories	ICD-9-CM diagnosis codes	Definition
^a^Chronic Kidney Disease (CKD)		
158	585, 5853, 5854, 5855, 5856, 5859	CKD
Cancer		
14	1530 1531 1532 1533 1534 1535 1536 1537 1538 1539 1590 2303 V1005	Colon Cancer
24	1740 1741 1742 1743 1744 1745 1746 1748 1749 1750 1759 2330 V103	Breast Cancer
19	1622 1623 1624 1625 1628 1629 2312 V1011	Lung Cancer
Stroke		
109	430 431 4320 4321 4329 43301 43311 43321 43331 43381 43391 4340 43400 43401 4341 43410 43411 4349 43490 43491 436	Acute cerebrovascular disease
112	4350 4351 4352 4353 4358 4359	Transient cerebral ischemia

^a^Persons receiving dialysis were excluded

Respondents younger than 21 years were excluded from this study. A total of 74,452 adults aged 21 years and older participated in the MEPS study during 2011–2013. Participants were further excluded if they had received dialysis or reported more than one of the studied conditions CKD, cancer and stroke simultaneously. Our study include a total of 52 sampled persons with non-dialysis dependent CKD. A total of 1104 sampled persons had CCS codes indicating stroke and 870 participants had CCS codes indicating colon, breast or bronchus/lung cancers (Table 1). Sampled persons without CKD, stroke or cancer totaled 72,241.

### Healthcare expenditures

Per-capita annual total direct health care expenditures, defined as third party (Medicare, Medicaid, private, other) and patient out-of-pocket payments for medical services were calculated. Types of medical services included inpatient, outpatient, prescription drug, dental, vision, home health, and payments for other medical equipment and services reported during the calendar years 2011–2013. Expenditure values were all calculated as year 2013 dollars. Out-of-pocket spending included self-reported payments for coinsurance and deductibles, and cash outlays for services, supplies, and other items not covered by health insurance. Out-of-pocket expenditures burden was calculated as the ratio of out-of-pocket spending to personal income and expressed as a percentage varying from 0 to 100 with high out-of-pocket spending burden as spending 10% or more of personal income on health care [19].

### Covariates

The panel of this study is composed of five rounds of interviews covering two full calendar years. Demographic questions were asked during each round of interview. Demographic variables including age, height and weight and race/ethnicity were self-reported and collected by MEPS. For this study, race/ethnicity was categorized as non-Hispanic White, non-Hispanic Black, Hispanic and other. Health insurance was categorized as public, private or none. Body mass index (BMI) was calculated as weight (kg) divided by height in m (squared). MEPS collected data on total family income and family size and five categories of poverty status were then defined by MEPS based on the ratio of family income to family size and composition: poor (less than 100% to the poverty line), near poor (100%-125% of the poverty line), low income (125%-less than 200% of the poverty line), middle income (200%- less than 400% of the poverty line) and high income (≥400% of the poverty line). Self-reported perceived mental and physical health statuses were ascertained in MEPS and categorized as excellent, very good, good, fair and poor. The presence of other co-occurring physical conditions were measured as binary variables based on self-reporting a physician diagnosis of angina, arthritis, asthma, coronary heart disease, high cholesterol, diabetes, emphysema, high blood pressure, heart attack, and other heart disease.

### Statistical analysis

All analyses accounted for the complex sampling design of MEPS dataset by using the sampling weight, variance estimation stratum and primary sampling unit. The Taylor-series approach was implemented to estimate standard errors for weighted survey estimates. Demographic characteristics, presence of other co-occurring physical conditions, total health care expenditures, out-of-pocket spending, and out-of-pocket spending burden was presented by condition categories using descriptive statistics. In this study, none of the participants with non-dialysis dependent CKD and a relatively small number of participants with cancer or stroke, had zero expenditures. Therefore the generalized linear model (glm) was used to estimate the adjusted total health care expenditures and out-of-pocket spending by the three condition categories compared to the group without CKD, cancer or stroke while simultaneously controlling for race/ethnicity, age, health insurance, gender, poverty status and presence of other co-occurring physical conditions.. We used the gamma distribution with log-link function and presented the marginal effects and their standard errors [20, 21]. The regression analyses did not adjust for perceived physical and mental health status and BMI due to the potential collinearity of these variables with the condition categories and other co-occurring physical conditions. All analyses were conducted using Stata 13 and *P* < 0.05 was considered statistically significant.

## Results

Table 2 demonstrates characteristics of the populations with the indicated conditions. The average age in groups with CKD, cancer, or stroke were 65.5, 66.1, and 68.2 years, respectively. Mean age in group without cancer, stroke or CKD was 47.8 years. Private insurance ranged from as low as 50.3% in the group with stroke to 65.2% in the group with CKD. Only 18.8% of the group with CKD rated their physical health as excellent or very good. In contrast, 39.7% and 25.0% of those with cancer and stroke, respectively, rated their physical health as excellent or very good. Table 3 demonstrates the prevalence of comorbidities in the three chronic condition groups, exclusively. Of the indicated conditions, the group with CKD had the highest prevalence of high cholesterol (85.0%), high blood pressure (87.8%) and diabetes (49.6%).

**Table 2:** Demographic and clinical characteristics of U.S. adults aged 21 years or older by condition categories, years 2011-2013

	Chronic Kidney Disease (CKD)^a^	Cancer^b^	Stroke	No CKD, cancer or stroke^c^
Study sample, *n* = 74,267	52	870	1104	72,241
Study population estimate over 3 year period, *N* = 940,337,592	607,506	9,206,890	10,520,699	650,226,082
Age (years)	65.5	66.1	68.2	47.8
Race				
Non-Hispanic White (%)	79.7	79.0	73.0	66.4
Non-Hispanic Black (%)	11.6	10.0	15.2	11.2
Hispanic (%)	7.4	8.0	7.8	14.8
Other (%)	1.3	3.0	4.0	7.6
Male (%)	48.7	15.8	49.1	48.5
BMI [kg/m (squared)]	28.3	26.7	26.9	27.1
Poverty Status^d^				
Poor (%)	9.3	10.9	19.5	12.1
Near Poor (%)	2.6	7.0	7.3	4.4
Low Income (%)	17.2	15.4	19.0	13.6
Middle Income (%)	32.8	30.6	30.4	30.3
High Income (%)	38.1	36.1	23.8	39.7
Health Insurance				
Public (%)	26.8	32.9	46.5	17.0
Private (%)	65.2	65.1	50.3	67.7
None (%)	8.1	2.0	3.2	15.4
Perceived Physical Health				
Excellent (%)	6.0	13.0	4.2	24.7
Very Good (%)	12.8	26.7	20.8	34.1
Good (%)	33.0	29.0	29.3	28.3
Fair (%)	35.3	16.8	25.6	9.6
Poor (%)	10.3	11.7	17.6	2.9

^a^Excludes dialysis patients

^b^Includes breast, colon & lung cancers

^c^Excludes CKD, cancer and/or stroke. The condition categories are mutually exclusive

^d^Based on the ratio of family income to family size and composition: poor = less than 100% to the poverty line, near poor =100%-125% of the poverty line, low income =125%-less than 200% of the poverty line, middle income =200%- less than 400% of the poverty line, and high income = ≥ 400% of the poverty line

**Table 3:** Prevalence of comorbidities among U.S. adults aged 21years or older by conditions, years 2011-2013

Comorbidity	CKD^a^	Cancer^b^	Stroke	No CKD, cancer or stroke^c^
Angina (%)	10.3	5.9	12.6	2.3
Arthritis (%)	63.7	55.2	61.2	25.8
Asthma (%)	11.8	14.7	11.8	9.3
Coronary Heart Disease (%)	20.8	12.7	26.6	5.3
High Cholesterol (%)	85.0	56.2	66.8	31.5
Diabetes (%)	49.6	19.3	31.2	9.3
Emphysema (%)	1.2	10.7	7.8	2.0
High Blood Pressure (%)	87.8	60.9	78.2	33.8
Heart Attack (%)	4.9	8.9	22.6	3.5
Other Heart Disease (%)	41.5	24.3	34.4	10.7

^a^Excludes dialysis patients

^b^Includes breast, colon & lung cancers

^c^Excludes CKD, cancer and/or stroke. The condition categories are mutually exclusive

**Fig. 1:**
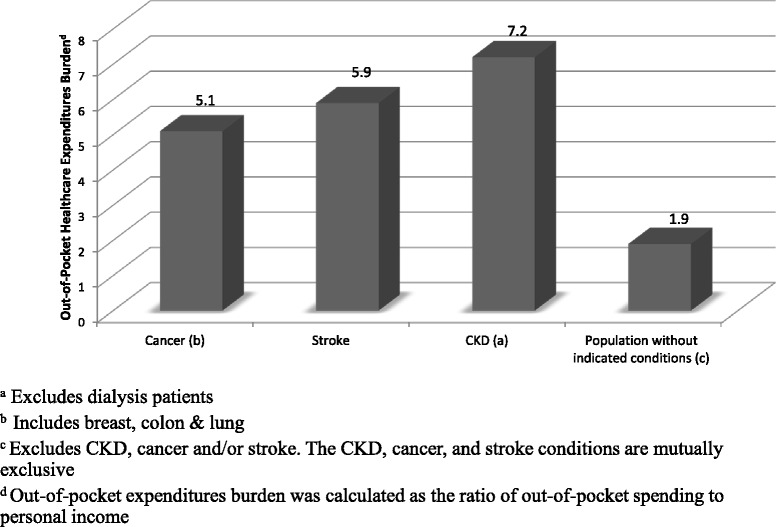
Mean out-of-pocket health care expenditures burden of U.S. adults aged 21 years or older by condition categories, years 2011–2013

The annual mean total healthcare expenditures ranged from as high as $12,877 for non-dialysis dependent CKD to as low as $7428 for the chronic condition cancer (Table 4). The annual mean out-of-pocket expenditures was also highest for the group with non-dialysis dependent CKD ($1439; IQR $688-$2732), and lowest for the group with stroke ($748; IQR $242–$1559). Fig. 1 shows the out-of-pocket health care expenditure burden, calculated as the ratio of out-of-pocket spending to personal income. The annual out-of-pocket health care expenditure burden for cancer and stroke were 5.1 and 5.8, respectively. However, the out-of-pocket healthcare expenditure burden for group with non-dialysis dependent CKD was 7.2, comparatively higher than the annual out-of-pocket health care expenditure burden for the other groups.

**Table 4:** Annual total direct and out-of-pocket healthcare expenditures (in $2013 US) of U.S. adults aged 21years or older by conditions, years 2011-2013

	Total direct expenditures ($)	Out-of-pocket expenditures ($)
	Median (IQR)	Median (IQR)
CKD^a^	12,877 (5031 – 19,710)	1439 (688 – 2732)
Cancer^b^	7428 (3460 – 18,323)	770 (349– 1703)
Stroke	8150 (3966– 19,375)	748 (242 – 1599)
Population without CKD, cancer or stroke^c^	$1189 (196 – 4388)	226 (20 – 764)

^a^Excludes dialysis patients; CKD, Chronic kidney disease

^b^Includes breast, colon & lung

^c^Excludes CKD, cancer and/or stroke. Condition categories are mutually exclusive

After adjusting for demographic variables and comorbidities, CKD, cancer and stroke were associated with significantly higher direct and out-of-pocket expenditures compared to the population without CKD, cancer or stroke. The highest difference in direct expenditures was noted in the group with cancer with $8608 (95% CI $6167-$11,049) higher total direct healthcare expenditures relative to population without CKD, cancer or stroke after adjustment for demographics and co-morbidities. The highest difference in out-of-pocket expenditures was noted in the group with CKD with $760 (95% CI 0-$1745) higher out-of-pocket spending relative to population without CKD, cancer or stroke after adjustment for demographics and co-morbidities (Table 5). In contrast, stroke was associated with $5992 (95% CI $4208-$7775) higher total direct healthcare expenditures and $246 (95% CI $87-$406) higher out-of-pocket expenditures relative to the group without CKD, cancer or stroke (Table 5).

**Table 5:** Adjusted differences in total direct and out-of-pocket health care expenditures (in $2013 US) of U.S. adults aged 21years or older by conditions, years 2011-2013

	^d^Differences in total direct healthcare expenditures ($)			^d^Differences in out-of-pocket expenditures ($)		
No CKD, cancer or stroke^c^	Referent	95% CI	*P-value*	Referent	95% CI	*P-value*
CKD^a^	$4746	$1775–$7718	0.002	$760	0–1745	0.130
Cancer^b^	$8608	$6167–$11,049	<0.001	$419	$158–$679	0.002
Stroke	$5992	$4208–$7775	<0.001	$246	$87–$406	0.002

^a^Excludes dialysis patients; CKD, chronic kidney disease

^b^Includes breast, colon & lung

^c^Excludes CKD, cancer and/or stroke. The condition categories are mutually exclusive

^d^Analysis compares expenditure per chronic condition category, adjusted for the variables listed and race/ethnicity, age, health insurance, gender, poverty status and presence of other co-occurring physical conditions and all costs were inflated to 2013 dollars

## Discussion

This study showed that adults with diagnosed non-dialysis dependent CKD based on 585 ICD9 codes had higher total direct and higher out-of-pocket healthcare expenditures compared to adults with stroke or cancer. Our analyses included adults from all stages of CKD including early, moderate, and advanced stages, who were aware of their condition or actively seeking treatment. We chose to compare non-dialysis dependent CKD healthcare expenditures with those for cancer and stroke due to the fact that these chronic conditions are among the most common, costly, and preventable chronic conditions in the U.S [18]. The adjusted out-of-pocket cost burden for the group with CKD was substantially higher compared to the out-of-pocket cost burden for adults without CKD, cancer or stroke.

Our findings were similar to a previous study that examined the annual total expenditures for year 2011 for patients with both CKD and diabetes [15]. However, CKD ascertainment was different between the two studies. In our study, we used the MPC files and associated ICD9 codes to define current conditions including the presence of CKD. In the Ozieh et al. article [15], respondents were identified as having CKD if they responded yes to the question “Has diabetes caused kidney problems?”, while diabetes was ascertained by answering yes to the question: “Have you ever been told by a doctor or health professional that you had diabetes?”

Progression of non-dialysis CKD is known to be associated with escalating healthcare costs [6, 8, 22]. In an a analysis of 83,705 adults with type 2 diabetes enrolled in a Medicare Advantage plan, Stage 4 CKD was associated with an incremental cost increase of $33,131 relative to stage 1 or no CKD in the 98^th^ percentile of total costs [22]. In a study of Medicare enrollees, CKD was estimated to increase the average Medicare payment for part A by a factor of 12.1 and for part B by a factor of 4.4 among men. Somewhat lower estimates were noted for female enrollees [18]. However, individuals receiving dialysis were not excluded. Previous studies did not examine out of pocket expenditures and associated out of pocket burden. Our study suggests that out of pocket healthcare expenditures for adults with non-dialysis dependent CKD may be higher than those for adults with cancer or stroke and lead to a higher financial burden for individual patients. These findings add to a growing body of literature that confirms the strong financial burden of healthcare costs for patients with CKD. The higher costs for CKD also hold public health significance because prevalence of CKD is increasing among Medicare Part A beneficiaries, while prevalence of stroke and many cancers are decreasing [9].

The unadjusted higher total healthcare expenditures for CKD may be due to the higher number of co-morbidities in this group. The group with CKD defined by 585 ICD9 codes had the highest prevalence of high cholesterol, diabetes, and high blood pressure. Higher number of co-morbidities is associated with higher number of physician visits and medications utilized as documented in previous studies [8, 11, 15]. In fact, the majority of adults with moderate to severe CKD may have 3 or more co-morbid conditions and take at least 5 medications [11]. The unadjusted median total and out-of-pocket healthcare expenditures of respondents with CKD were substantially larger than expenditures for respondents with cancer and stroke. However, total direct healthcare expenditures decreased and became more comparable to those for stroke and cancer after adjusting for demographic variables and comorbidities. The out-of-pocket expenditures remained higher in the CKD group.

Results from our study are generalizable to adults who are aware of their conditions or who visited a healthcare provider for the condition during the study period. Our CKD sample is therefore, not representative of all U.S. adults with CKD, because the majority with CKD remain unaware of their condition [23].

The strengths of this study included the use of a nationally representative survey of U.S. healthcare expenditures and MEPS data are weighted so that expenditure estimates for a given condition reflect expenditures for the total U.S. population with that condition. However, as noted earlier, presence of conditions in MEPS are based on self-report and rely on accurate recollection by respondents. Also, records in the MEPS Conditions File correspond to current conditions; meaning, the respondent reported the condition as the reason for a particular medical event such as an outpatient visit, the reason for one or more episodes of disability days, or ‘bothering’ the person during the reference period., The number of sampled persons with non-dialysis dependent CKD with 585 ICD9 codes was small and confidence intervals for the healthcare expenditure estimates were wider than the estimates for the other conditions. Therefore, results from our study should be interpreted in light of the small number of respondents with non-dialysis dependent CKD included in the analyses. The MEPS is representative of the U.S. population and our findings may not be generalizable to non-U.S. populations. Differences in out-of-pocket expenditures for this group relative to the group without CKD, cancer or stroke included zero, which may have been due to the small sample size. Because many adults with CKD remain unaware of their condition, the results from this study do not reflect healthcare expenditures for all U.S. adults with CKD.

## Conclusions

In conclusion, our findings in adults with diagnosed CKD showed that CKD was associated with both high total and out of pocket healthcare expenditures in the U.S. and these expenditures were similar to those of other costly chronic diseases, namely cancer and stroke. Future research should examine interventions for preventing the onset or progression of CKD and reducing the out-of-pocket expenditure burden for adults with CKD.

## Additional file

Additional file 1: Relevant Stata commands used in the study available in a Stata commands ‘do’ file. (DO 12 kb)

## Abbreviations

BMI: Body mass index
CCC: Clinical classification codes
CKD: Chronic kidney disease
ICD-9-CM: International classification of diseases, 9^th^ Edition, clinical modification
MEPS: Medical expenditures panel survey
